# Comparing the predictive accuracy of life’s essential 8 and life’s crucial 9 scores for all-cause mortality in COPD patients among US adults: a prospective cohort study

**DOI:** 10.1186/s12889-026-26333-4

**Published:** 2026-01-22

**Authors:** Shuhui Qiu, Yiyi Chang, Can Cui, Feng Wang, Xianyan Sun, Dapeng Li

**Affiliations:** 1 Department of Respiratory and Critical Care Medicine, The First Hospital of Jilin University, Changchun, Jilin China; 2 Department of General Practice, The First Hospital of Jilin University, Changchun, Jilin China; 3 Department of Neurosurgery, The First Hospital of Jilin University, Changchun, Jilin China

**Keywords:** COPD, Cardiovascular health, Life’s essential 8, Life’s crucial 9, NHANES, Cohort study

## Abstract

**Background:**

Life’s Essential 8 (LE8) represents an established score for quantifying cardiovascular health (CVH). Life’s Crucial 9 (LC9) is a newly proposed metric that integrates psychological health with LE8. The prognostic significance of these scores concerning all-cause mortality risk in COPD patients remains unclear.

**Methods:**

We analyzed data from NHANES 2005–2018 to examine the relationship between CVH (quantified by LC9 and LE8 scores) and all-cause mortality among adults with COPD. Cox proportional hazards regression was applied, and additional analyses were conducted to assess robustness.

**Results:**

Among 1,757 participants (380 deaths, 21.63%) over a 72-month median follow-up, the highest LC9 score quartile was associated with a significantly lower mortality risk when compared to the lowest quartile (HR = 0.36, 95% CI: 0.22–0.59). A similar inverse association was observed for the LE8 score. LC9 consistently demonstrated marginally higher predictive accuracy than LE8 across analyses.

**Conclusion:**

Higher CVH levels defined by either LC9 or LE8 were strongly associated with reduced all-cause mortality in COPD patients. The slightly superior performance of LC9 highlights the added value of psychological health inclusion into cardiovascular health assessment to enhance mortality risk stratification and guide comprehensive COPD management.

**Supplementary Information:**

The online version contains supplementary material available at 10.1186/s12889-026-26333-4.

## Introduction

Chronic obstructive pulmonary disease (COPD) is marked by persistent inflammation of the airways, leading to sustained limitation of airflow [1, 2], that may occur across all age groups but predominantly affects individuals aged over 40 years [3]. According to the World Health Organization, COPD caused an estimated 3.23 million deaths in 2019, accounting for it being ranked as the third most common global cause of mortality [4]. By 2040, it is projected that COPD would become the fourth key cause of premature death, exerting an enormous burden on the global economy and public health [5]. COPD is characterized by an indolent evolution and systemic effects, and frequently co-occurs with comorbidities like cardiovascular disease (CVD) and depression [6]. Studies has indicated individuals diagnosed with COPD are face a greater risk of CVD relative to those without the condition [7], and the presence of CVD increased morbidity and mortality risk in COPD patients [8, 9]. Similarly, depression was significantly more prevalent in COPD patients relative to non-COPD individuals, with comorbid depression further elevating the COPD mortality [9, 10]. Therefore, the identification and management of comorbidities might have a significant impact on outcomes of COPD.

The concept of cardiovascular health (CVH) was formally proposed by the American Heart Association (AHA) in 2010, emphasizing how preventing CVD important [11]. To increase the sensitivity of CVH assessment to individual variations, the AHA introduced Life’s Essential 8 (LE8) in 2022 as a refined metric [12]. Existing evidence repeatedly indicated that LE8 was linked to several health outcomes, including cardiovascular events, non-alcoholic fatty liver disease, and all-cause mortality [13–15]. LE8 had been utilized to predict the risk of multiple non-communicable chronic diseases, such as COPD [16]. The AHA also stressed the need of sustaining CVH in the context of psychological health [12]. A novel composite metric, termed Life’s Crucial 9 (LC9), has been introduced in recent academic work. It merges the LE8 framework with a psychological health component [17]. Research indicated LC9 was linked to all-cause mortality [18]. However, limited evidence is available regarding the relationship of LC9 or LE8 with death risk among COPD patients. And no studies had compared the prognostic ability of LC9 and LE8 for COPD-related all-cause death.

To fill this knowledge vacuum, we analyzed data from a sizable NHANES cohort of adults aged 40 and older. Specifically, we examined how CVH—evaluated using both the LC9 and LE8 scores—was associated with all-cause mortality among patients with COPD.

## Methods

### Study design and data source

With a focus on the years 2005–2018, our research analyzed data from the US NHANES. The main goal was to investigate relationships between CVH (LC9 or LE8) and all-cause death in community-dwelling persons with COPD.

The National Center for Health Statistics (NCHS), a division of the CDC in the US, gathers data for the NHANES database. This study seeks to evaluate the nutritional status and overall health profile of the nationwide population. The representativeness of the data for the US population is guaranteed by a complex, multistage design in survey techniques, which excludes people in institutional settings. The data is made accessible by the NCHS for use by researchers.

Participants in the NHANES survey are first interviewed at home before being invited to a mobile examination center (MEC) for thorough health examination. Physical measurements, specialist examinations, and lab evaluations are all included in the examination. For the NHANES survey, it should be noted that each participant gave written informed consent [19]. The NHANES website provides public access to the dataset [20].

### Study population selection

**Fig. 1:**
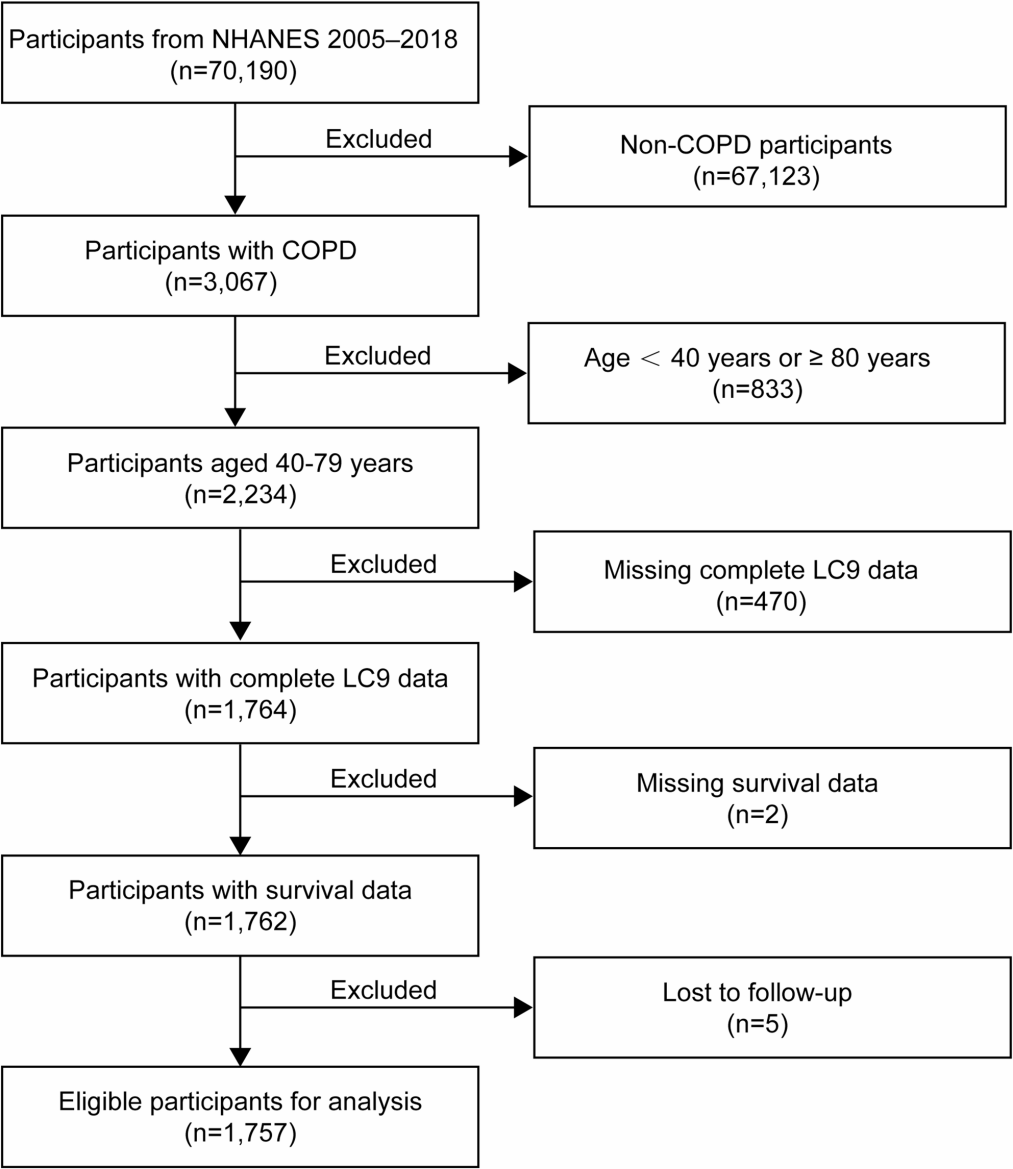
Flowchart for this cohort study in NHANES 2005–2018. NHANES, National Health and Nutrition Examination Survey; COPD, Chronic Obstructive Pulmonary Disease; LC9, Life’s Crucial 9

In the 2005–2018 NHANES dataset, community-dwelling persons aged from 40 to 79 who were diagnosed with COPD by doctors were enrolled in our study. COPD was identified utilizing NHANES questionnaire data based on self-reported physician diagnoses of COPD, chronic bronchitis, or emphysema. Several earlier studies based on NHANES data have successfully identified COPD patients using this strategy [21, 22]. After excluding individuals without CVH data, survival data, or confounders, as well as those who were unable to receive a COPD diagnosis, 1,757 of the original 70,190 participants remained in the study. The screening procedure for the sample, which is representative of 10,649,797 United States citizens, is shown in Fig. 1.

### Measurement of CVH scores

LE8 is defined by eight components: diet, physical activity, nicotine exposure, sleep, BMI, non-HDL cholesterol, blood glucose, and blood pressure, each with established scoring methods [12]. The LE8 score, derived via a modified Delphi process where experts rated eight CVH indicators (0–100 points), is the average of these components. Its extension, the LC9, incorporates psychological health (depression score) as an additional, equally weighted component, with the total score being the average of all nine metrics.

Comprehensive scoring criteria for all components have been published [18, 23] (Supplementary Table 1). Dietary scores were derived by applying the Healthy Eating Index-2015 to data from two 24-hour dietary recalls [24, 25]. Data on physical activity, nicotine exposure, sleep, diabetes history, and medication use came from validated questionnaires, while physical examinations provided anthropometric (for BMI) and blood pressure measurements. Blood tests measured lipids, fasting glucose, and HbA1c. Depression was evaluated using the Patient Health Questionnaire-9 (PHQ-9) [26]. Its scores were linearly converted to a 0–100 scale, with a score of ≥ 5 (a common clinical cut-point in chronic diseases) indicating depressive symptoms [27, 28].

LC9/10 or LE8/10 means that the LC9 or LE8 score is divided by 10.

### Mortality data collection

Using NHANES 2005–2018, we longitudinally linked the data with mortality data from the National Death Index (NDI), with follow-up continuing until December 31, 2019. All-cause mortality, or deaths from any cause, was the outcome that our study assessed.

### Covariates

Covariates were selected based on prior literature and clinical expertise, consistent with standard practice in observational epidemiology. Collected demographic data included age (grouped as 40–49, 50–59, 60–69, 70–79 years), gender, race/ethnicity (Non-Hispanic Black, Non-Hispanic White, Mexican American, Other), marital status, education level, and smoking status (defined as never [< 100 cigarettes lifetime], former, or current).

Diabetes mellitus (DM) was defined by self-reported insulin use, physician diagnosis, or use of hypoglycemic agents. Hypertension required a confirmed diagnosis, antihypertensive medication use, or an average blood pressure ≥ 140/90 mmHg across three readings. A history of CVD was based on a physician’s diagnosis of stroke, myocardial infarction, angina, or coronary heart disease. Metabolic syndrome (MetS) was defined as meeting ≥ 3 of the following criteria: waist circumference ≥ 102 cm (men)/≥88 cm (women); HDL < 40 mg/dL (men)/<50 mg/dL (women); triglycerides ≥ 150 mg/dL; fasting glucose ≥ 110 mg/dL; blood pressure ≥ 130/≥85 mmHg. Depression was assessed using the PHQ-9.

### Statistical analyses

Analyses were conducted on complete cases after excluding participants with missing data. In accordance with NHANES guidelines, we applied sample weights (MEC examination weights) along with stratification (SDMVSTRA) and clustering (SDMVPSU) variables to account for the complex survey design. Categorical and normally distributed continuous variables are presented as frequencies (percentages) and means ± standard deviations, respectively. Group comparisons were performed using Pearson’s chi-square tests and ANOVA.

Our analytic approach was consistent with recent NHANES LE8 implementations [29]. The association of CVH and PHQ-9 scores with all-cause mortality was evaluated using weighted Cox proportional hazards regression, sequentially adjusted for demographic factors (Model 1) and clinical comorbidities (Model 2). Non-linear relationships were explored with weighted restricted cubic splines. Model predictive accuracy was assessed via the bootstrap-corrected C-index, with net reclassification improvement (NRI) and integrated discrimination improvement (IDI) quantifying incremental value. Time-dependent AUC curves compared LC9 and LE8 over time. Sensitivity and subgroup analyses tested robustness, and all models satisfied goodness-of-fit criteria. Analyses were performed in R 4.2.0, with a two-sided p-value < 0.05 considered statistically significant.

## Results

### Basic characteristics of participants

Table 1 summarizes the baseline characteristics of the study participants. There were 1,757 participants in all, most of whom were aged 60 years or older (58.72%), female (60.78%), and non-Hispanic white (79.22%). Mean LE8 and LC9 scores were 57.85 (0.51) and 59.89 (0.49), respectively. Participants were stratified into quartiles of LC9/10: Q1 (*n* = 428), Q2 (*n* = 438), Q3 (*n* = 463), and Q4 (*n* = 428). Higher LC9 scores were associated with older age, non-Hispanic white ethnicity, being married or cohabiting, higher education, and non-smoking status. Over a median follow-up of 72 months (interquartile range: 36–120), 380 deaths (21.63%) occurred. Higher CVH scores exhibited an inverse association with all-cause mortality (Tables 2 and 3).

**Table 1:** Baseline characteristics of study population by life’s crucial 9/10 quartiles

Characteristics	Overall	Q1	Q2	Q3	Q4	*p* -value
	(*n* = 1,757)	(*n* = 428)	(*n* = 438)	(*n* = 463)	(*n* = 428)	
LE8 score	57.85(0.51)	37.87(0.57)	50.72(0.33)	60.52(0.28)	73.92(0.45)	< 0.0001
LC9 score	59.89(0.49)	38.97(0.49)	53.06(0.18)	62.87(0.20)	76.02(0.42)	< 0.0001
Age(years), n (%)						0.12
40–49	317(20.94)	82(22.68)	65(18.78)	90(23.01)	80(19.57)	
50–59	448(28.34)	128(33.80)	114(27.67)	105(27.86)	101(25.73)	
60–69	578(31.62)	147(30.35)	157(36.96)	146(27.80)	128(31.88)	
70–79	414(19.10)	71(13.17)	102(16.59)	122(21.34)	119(22.82)	
Sex, n (%)						0.16
Female	1,014(60.78)	261(62.56)	258(63.46)	253(54.87)	242(63.05)	
Male	743(39.22)	167(37.44)	180(36.54)	210(45.13)	186(36.95)	
Race, n (%)						0.01
Non-Hispanic White	1,037(79.22)	223(73.10)	253(78.34)	279(79.24)	282(83.87)	
Non-Hispanic Black	356(8.73)	119(13.68)	100(10.98)	76(6.88)	61(5.49)	
Mexican American	108(2.27)	22(2.49)	24(1.76)	39(3.02)	23(1.81)	
Others	256(9.79)	64(10.73)	61(8.92)	69(10.87)	62(8.83)	
Marriage, n (%)						< 0.001
Married/Living with partner	910(59.34)	196(49.78)	197(52.34)	252(60.71)	265(69.72)	
Widowed/Divorced/Separated	679(32.95)	182(40.38)	203(40.29)	169(30.90)	125(24.34)	
Never married	168(7.71)	50(9.84)	38(7.37)	42(8.40)	38(5.94)	
Educational attainment, n (%)						< 0.0001
< High school	473(18.90)	156(31.36)	133(22.25)	116(16.33)	68(10.56)	
High school	461(28.31)	108(31.28)	125(30.24)	118(27.32)	110(25.82)	
> High school	823(52.79)	164(37.36)	180(47.50)	229(56.36)	250(63.62)	
Smoking status, n (%)						< 0.0001
Former	472(27.89)	50(10.06)	91(20.64)	152(31.39)	179(41.86)	
Current	640(35.89)	240(58.81)	187(45.77)	145(32.84)	68(16.16)	
Never	645(36.22)	138(31.13)	160(33.59)	166(35.77)	181(41.98)	
Diabetes, n (%)						< 0.0001
Yes	520(25.31)	229(46.08)	147(32.88)	98(18.91)	46(11.88)	
No	1,237(74.69)	199(53.92)	291(67.12)	365(81.09)	382(88.12)	
Hypertension, n (%)						< 0.0001
Yes	1,157(60.54)	342(75.63)	313(68.61)	297(61.60)	205(43.50)	
No	600(39.46)	86(24.37)	125(31.39)	166(38.40)	223(56.50)	
CVD, n (%)						< 0.0001
Yes	558(28.06)	197(42.80)	157(34.85)	122(25.20)	82(15.88)	
No	1,199(71.94)	231(57.20)	281(65.15)	341(74.80)	346(84.12)	
MetS, n (%)						< 0.0001
Yes	796(43.56)	271(62.63)	232(53.84)	197(45.89)	96(21.04)	
No	961(56.44)	157(37.37)	206(46.16)	266(54.11)	332(78.96)	
Chronic bronchitis, n (%)						< 0.0001
Yes	499(26.33)	151(35.79)	139(31.89)	120(25.40)	89(17.30)	
No	1249(73.08)	274(64.21)	296(68.11)	342(74.60)	337(82.70)	
Emphysema, n (%)						< 0.001
Yes	1290(74.88)	307(70.78)	308(70.05)	337(73.60)	338(83.17)	
No	462(24.86)	119(29.22)	130(29.95)	126(26.40)	87(16.83)	

Continuous variables were presented as means (standard error). Categorical variables were expressed as counts (percentages)

Q1-Q4 indicates quartile 1-quartile 4

### Associations of CVH scores or PHQ-9 score with all-cause mortality

In every model, there was a substantial correlation between lower risk of all-cause death and both LC9 and LE8 scores. In contrast to the lowest LC9 quartile (Q1), adjusted HRs in the fully adjusted model (model 2) were 0.78 (95% CI, 0.54–1.13; *p* = 0.19) for Q2, 0.57 (95% CI, 0.38–0.85; *p* = 0.01) for Q3, and 0.36 (95% CI, 0.22–0.59; *p* < 0.0001) for Q4. For LE8, adjusted HRs dropped from 0.86 (95% CI, 0.65–1.15; *p* = 0.32) for Q2 to 0.72 (95% CI, 0.45–1.13; *p* = 0.15) for Q3, and 0.42 (95% CI, 0.26–0.67; *p* < 0.001) for Q4 in model 2. Detailed results are shown in Tables 2 and 3. Unweighted Cox regression showed consistent direction and significance for correlations between LC9 or LE8 scores and all-cause death (Supplementary Tables 2 and 3).

Additionally, in model 2, the PHQ-9 score was substantially linked to all-cause mortality risk, regardless of whether it was considered a continuous or categorical variable (Supplementary Table 4).

**Table 2:** Relationship between life’s crucial 9 score and all-cause mortality in COPD patients

	Crude Model		Model 1		Model 2	
	HR(95% CI)	*p*	HR(95% CI)	*p*	HR(95% CI)	*p*
LC9/10	0.76(0.69,0.83)	< 0.0001	0.73(0.66,0.80)	< 0.0001	0.74(0.66,0.83)	< 0.0001
Q1 [15.00, 48.33]	Ref		Ref		Ref	
Q2 (48.33, 58.33]	0.85(0.60,1.21)	0.37	0.75(0.52,1.06)	0.10	0.78(0.54,1.13)	0.19
Q3 (58.33, 67.78]	0.62(0.43,0.89)	0.01	0.53(0.37,0.76)	< 0.001	0.57(0.38,0.85)	0.01
Q4 (67.78, 97.22]	0.36(0.24,0.55)	< 0.0001	0.32(0.21,0.48)	< 0.0001	0.36(0.22,0.59)	< 0.0001
p for trend		< 0.0001		< 0.0001		< 0.0001

Crude Model: unadjusted

*HR* hazard ratio, *95% CI* 95% confidence interval

Model 1: adjusted for age, sex, race, marriage, education, and smoking status

Model 2: adjusted for all covariates

**Table 3:** Relationship between life’s essential 8 score and all-cause mortality in COPD patients

	Crude Model		Model 1			Model 2	
	HR(95% CI)	*p*	HR(95% CI)	*p*		HR(95% CI)	*p*
LE8/10	0.78(0.70,0.85)	< 0.0001	0.77(0.70,0.84)		< 0.0001	0.79(0.70,0.88)	< 0.0001
Q1 [11.25, 46.25]	Ref		Ref			Ref	
Q2 (46.25, 56.25]	0.79(0.58,1.07)	0.13	0.82(0.63,1.07)		0.15	0.86(0.65,1.15)	0.32
Q3 (56.25, 66.25]	0.64(0.41,1.01)	0.05	0.64(0.43,0.95)		0.03	0.72(0.45,1.13)	0.15
Q4(66.25,100.00]	0.36(0.24,0.54)	< 0.0001	0.37(0.25,0.54)		< 0.0001	0.42(0.26,0.67)	< 0.001
p for trend		< 0.0001			< 0.0001		< 0.0001

Crude Model: unadjusted

Model 1: adjusted for age, sex, race, marriage, education, and smoking status

Model 2: adjusted for all covariates

### Restricted cubic splines

**Fig. 2:**
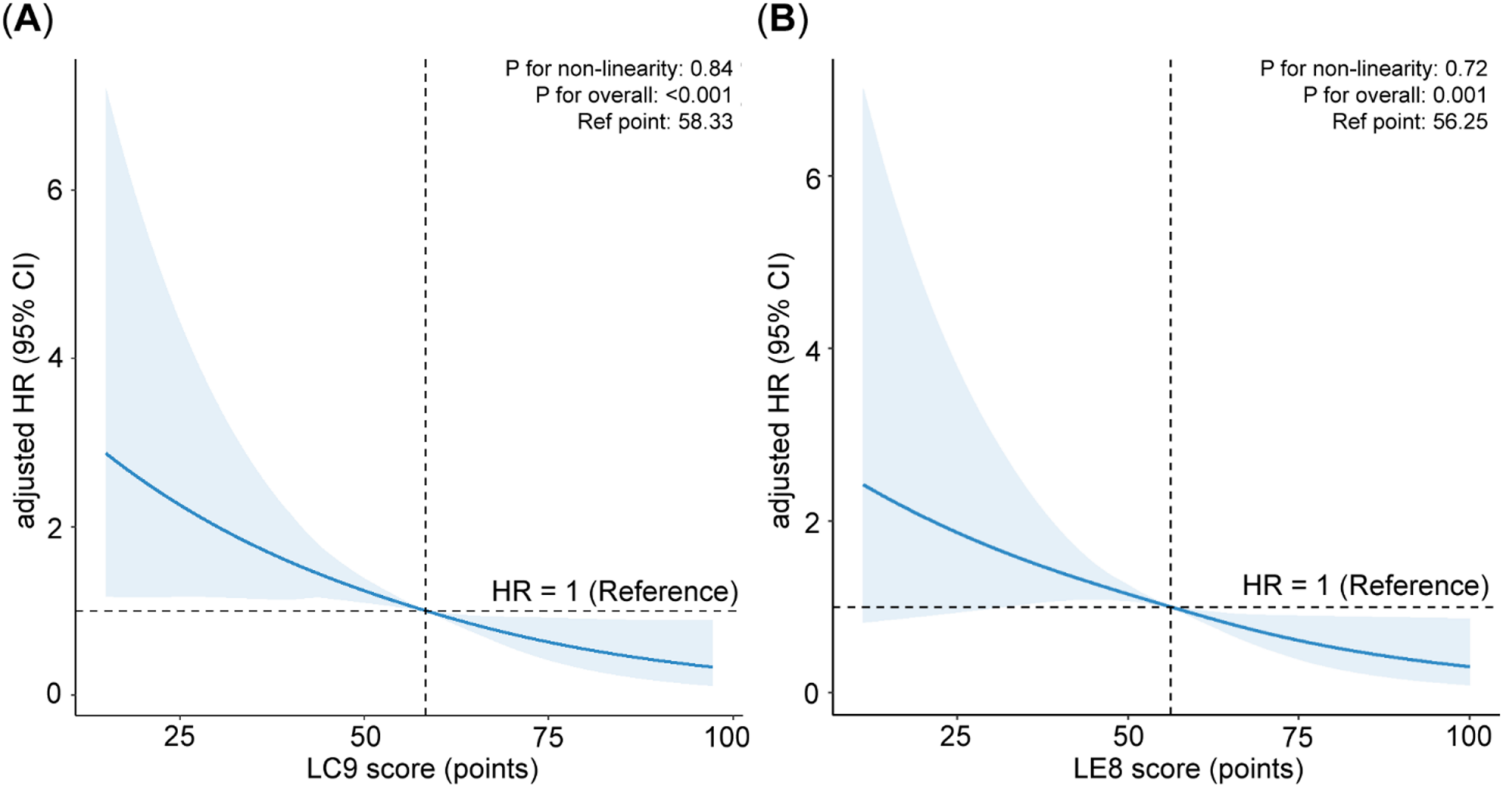
The RCS curves of the relationships of cardiovascular health scores with all-cause mortality in COPD patients after adjusting all covariates (**a**) Life’s Crucial 9 score (**b**) Life’s Essential 8 score

No non-linear connection found between CVH scores (LC9 and LE8) and mortality in the completely adjusted model (LC9: p for non-linearity = 0.84; LE8: p for non-linearity = 0.72) (Fig. 2). A 26% decrease in mortality risk was linked to every 10-point rise in LC9 (reference 58.33) (HR = 0.74, 95% CI: 0.66–0.83; p for overall < 0.001). Similarly, there was a 21% decrease in death risk for every 10-point rise in LE8 (reference 56.25) (HR = 0.79, 95% CI: 0.60–0.88; p for overall = 0.001).

### Comparison of predictive performance: LC9 vs. LE8 for all-cause mortality

LC9 showed slightly better discriminative ability than LE8 for all-cause death in COPD patients, with a marginally higher C-index (0.774 vs. 0.770) and an improvement of 0.0049 (95% CI, 0.0018–0.0091; *p* < 0.001). While the NRI was 12.8% (95% CI, 3.8% − 19.9%; *p* = 0.01) and the IDI was 0.005 (95% CI, 0.001–0.012; *p* = 0.03) (Table 4).

**Table 4:** Improvement in discrimination and reclassification of life’s crucial 9 compared with life’s essential 8

Model	△C index (95%CI)	*p*	NRI (95%CI)	*p*	IDI (95%CI)	*p*
Base model +LE8/10	Ref	Ref	Ref	Ref	Ref	Ref
Base model +LC9/10	0.0049 (0.0018, 0.0091)	< 0.001	12.8% (3.8%, 19.9%)	0.01	0.005 (0.001, 0.012)	0.03

Base model included age, sex, race, marriage, education, smoking status, diabetes, hypertension, CVD, and MetS

ΔC index, difference in the concordance index

The time-dependent AUC curves demonstrated that the LC9 score was consistently marginally more accurate than the LE8 score in predicting all-cause death among patients with COPD across all follow-up intervals in the fully adjusted model (Supplementary Fig. 1).

### Subgroup analyses and sensitivity analyses

**Fig. 3:**
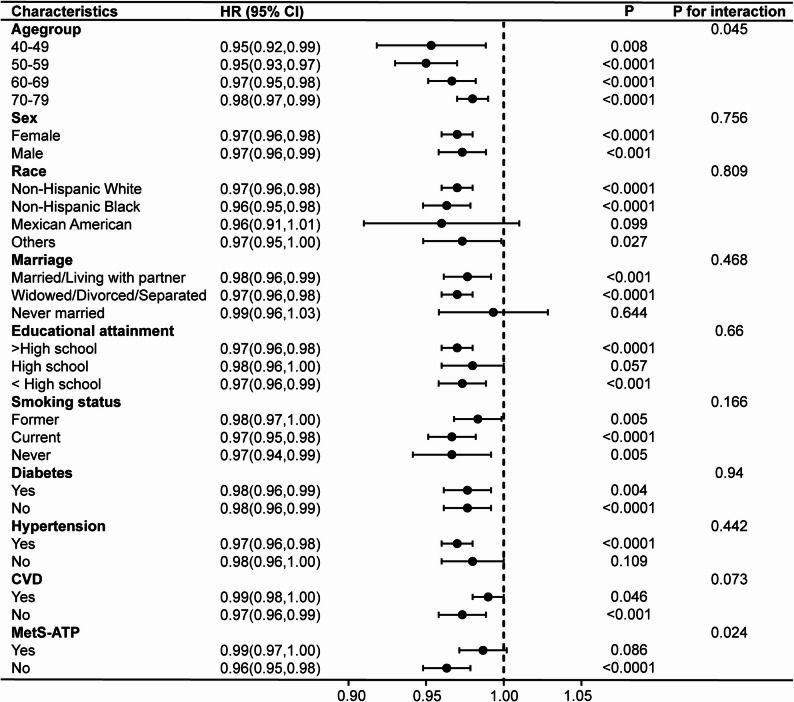
Subgroup analyses of the relationships between Life’s Crucial 9 and all-cause mortality in COPD patients

**Fig. 4:**
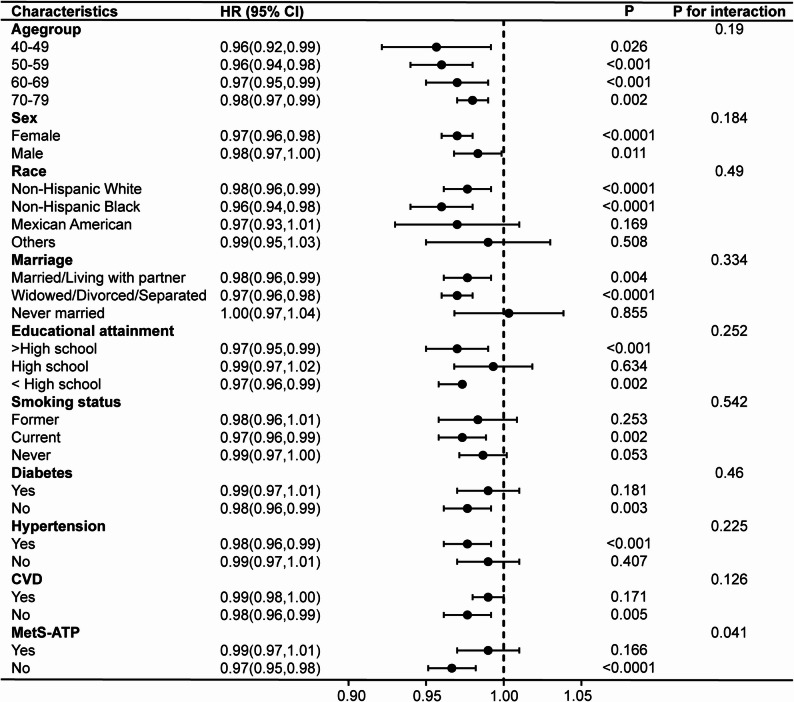
Subgroup analyses of the relationships between Life’s Essential 8 and all-cause mortality in COPD patients

To assess differences across subgroups, subgroup analyses and interaction tests were performed on the basis of the fully adjusted model (Figs. 3 and 4). LC9 significantly interacted with age and MetS, while LE8 interacted with MetS regarding all-cause death of COPD (*p* for interaction < 0.05). In participants without MetS, LC9 and LE8 both shown stronger correlations with risk of mortality (HR = 0.96, 95% CI: 0.95–0.98, *p* < 0.0001; HR = 0.97, 95% CI: 0.95–0.98, *p* < 0.0001, respectively). Stratified Cox regressions were performed for LC9 by age group and by presence of MetS, and for LE8 by presence of MetS; specific results are given in Supplementary Table 7.

The robustness of our outcomes was confirmed by consistent Cox regression results from sensitivity analyses that excluded participants whose follow-up was less than 24 months (Supplementary Table 8) and less than 36 months (Supplementary Table 9).

## Discussion

The current study showed that, after adjusting multiple covariates, the relationships between LC9 or LE8 score and all-cause death among people with COPD were statistically significant in a representative group of US adults, using NHANES data. Moreover, the depression score was significantly correlated with mortality risk of people with COPD. Our outcomes also showed that, after incorporating the depression score, the LC9 score predicted mortality with slightly greater accuracy than the LE8 score, although the magnitude of this improvement was modest. To the best of all that we know, this is the first research to evaluate the prognostic efficacy of LC9 relative to LE8 and to assess the association between the novel LC9 CVH score and mortality from all causes in US adults with COPD. Notably, the completely modified model included variables such as hypertension, diabetes, CVD, and MetS. Given that these disease states may serve as mediators in the pathway through which CVH influences mortality, this model may underestimate the total effect of CVH and instead reflect its direct or residual effect independent of these common comorbidities. Nevertheless, this analytic approach demonstrates that even after accounting for these clinical conditions, CVH scores still provide independent prognostic information, underscoring the importance of adopting comprehensive health management strategies rather than focusing solely on single-disease management for individuals with COPD.

CVH is an overall metric reflecting individual health behaviors and health factors. Globally, CVH levels have been closely associated with the incidence and mortality of CVD [13, 30] and have additionally been linked to various conditions that predispose individuals to CVD [16]. According to a prospective cohort research with 170,726 individuals from the UK, improving LE8-based CVH scores may lower the risk of several prevalent non-communicable diseases, including COPD, cancer, incident metabolic diseases, and mental and behavioral disorders [16]. This trend was further confirmed in the American population, where research revealed that higher LE8 scores were non-linearly and independently linked to decreased rates of non-alcoholic fatty liver disease (NAFLD) [15]. Additionally, a prospective cohort study in China demonstrated that among young and middle-aged adults, higher LE8 scores were inversely associated with the risks of cardiovascular disease (CVD) and all-cause mortality [31]. Recent researches have also shown that LE8 scores are inversely correlated with mortality from all causes in COPS patients [32, 33]. Our findings echo previous results, showing that individuals with higher CVH scores, as reflected by higher LC9 or LE8 scores, had lower all-cause death risk of COPD. This finding was still robust even after controlling for a number of potential confounding variables, which significantly enhancing the existing knowledge base. At the same time, we acknowledge that self-reported physician diagnosis may include overlap with asthma or chronic bronchitis and miss undiagnosed COPD, which likely biases associations toward the null. To assess robustness, we established stricter COPD diagnostic criteria based on existing literature [34, 35], which refer to meeting any of the following: (1) doctor told you have COPD; (2) FEV1/FVC < 0.7 following inhalation of a bronchodilator; (3) doctor told you have emphysema and former or current smoking; (4) individuals over 40 years old, with a smoking or chronic bronchitis history, and receiving COPD treatment including inhaled corticosteroids, mast cell stabilizers, leukotriene modifiers, or PDE-4 inhibitors. Sensitivity analyses were conducted for participants meeting stricter COPD diagnostic, yielding results consistent with the main findings (Supplementary Tables 5 and Supplementary Table 6), thereby validating the robustness of the study’s main outcomes.

Previous studies have demonstrated that depression, a common comorbidity in COPD, is closely correlated with mortality in COPD patients [9, 10, 36]. According to a systematic review and meta-analysis, people with COPD and comorbid depression were 83% more likely to die than those without depression [10]. Interestingly, a retrospective multicenter study found that mental health service utilization among COPD patients was associated with a 30% lower mortality risk compared to primary care alone [36]. Additional research has indicated that depression is an independent predictor of mortality among individuals with COPD [37]. Consistent with previous findings, our results demonstrated an independent positive correlation between PHQ-9 scores and mortality risk among individuals with COPD. However, for PHQ-9 scores of 10 or greater, a well-established threshold frequently employed in depression clinical research [26], our Cox regression analysis did not reach statistical significance (HR = 1.21, 95% CI: 0.87–1.68, *p* = 0.25). We identified two possible reasons for this finding: first, the sample size with PHQ-9 ≥ 10 was limited, resulting in low statistical power; second, we speculate that in this specific COPD population, mortality risk may be more sensitive to depressive symptoms, such that patients are affected even without reaching the conventional threshold for “moderate-to-severe depression” (PHQ-9 ≥ 10) due to their substantial physiological and psychological burden. Based on evidence from studies of other chronic somatic diseases (e.g., heart failure, diabetes) and their association with depression, we further explored using PHQ-9 ≥ 5 (representing the presence of depressive symptoms) as a dichotomous variable, and found that it had a strong correlation with death from all causes in COPD (HR = 1.70, 95% CI: 1.28–2.27, *p* < 0.001). Therefore, in COPD population, conceptualizing depression as a continuous risk spectrum starting from mild symptoms, rather than focusing solely on moderate-to-severe depression, may provide greater predictive value.

Based on existing evidence regarding the associations among CVD, depression, and COPD, we examined the potential links between CVH and COPD, attributing these associations to shared risk factors and underlying pathophysiological mechanisms. It is widely recognized that the shared pathophysiological mechanisms among these conditions primarily include hypoxia, systemic inflammation, and oxidative stress (OS) [38]. From the perspective of CVH components, smoking represents a common risk factor for CVD, depression, and COPD, increasing systemic inflammatory reactions and the production of reactive oxygen species (ROS) [10, 39], thereby contributing to higher risk of death for those who have COPD [40]. Physical activity was linked with lower death rates in patients with COPD [41], potentially due to increased pulmonary carbon monoxide diffusion, enhanced expiratory muscle strength, increased oxygen uptake, and reduced levels of inflammatory markers such as tumor necrosis factor-α and C-reactive protein [42]. The literature suggests that dietary patterns (e.g., high fruit/vegetable intake) and components (e.g., low sodium) may ameliorate COPD pathophysiology by modulating oxidant-antioxidant balance, inflammation, and respiratory function [43–45], providing protective effects against COPD mortality [45]. Insomnia was a condition affecting half of COPD patients and was partly related to crippling exhaustion and a progressive loss of function in these individuals [46]. Poor sleep has been linked to a higher mortality, according to research [47]. For COPD patients, low BMI was thought to be a separate risk indicator for death [48]. Studies indicated that both BMI < 20 kg/m² and BMI ≥ 40 kg/m² were linked to higher risk of death, while overweight COPD patients had the highest survival rates [49]. The observed protective effect of high BMI against COPD mortality should be interpreted with caution, as it may be nuanced by muscle mass and exercise capacity rather than adiposity alone, calling for further research on specific body composition metrics [50]. Currently, a small number of direct researches have looked at the relationships of COPD with lipid levels, blood glucose, and blood pressure. Existing study has demonstrated that a higher incidence of COPD is linked to higher levels of the ratio of non-HDL-C to HDL-C [51]. Additionally, DM has been linked to a 70% increase in all-cause death risk among COPD patients over a three-year period, potentially due to exacerbated systemic inflammation, as shown by increased levels of inflammatory markers such as (e.g., CRP) [52]. Both diastolic blood pressure < 80 or ≥ 90 mmHg and systolic blood pressure < 120 or ≥ 140 mmHg were associated with an increased risk of all-cause mortality in COPD patients [53]. Thus, the relationship between CVH and COPD was supported by various factors, including environmental exposures, inflammatory pathways, oxidative stress, and overlapping metabolic conditions, highlighting the necessity for a comprehensive health management approach. In addition to the biological pathways linking CVH to COPD outcomes, it is also important to contextualize our findings in relation to established COPD prognostic models. Traditional measures such as the BODE (Body mass index, airflow Obstruction, Dyspnea, and Exercise capacity) and ADO (Age, Dyspnea, airflow Obstruction) scores primarily reflect physiological impairment and symptom severity. In contrast, the CVH score emphasizes modifiable lifestyle factors and cardiometabolic health, offering a complementary dimension of risk stratification. Rather than replacing established prognostic tools, CVH may provide additional insight into prevention-oriented risk modification, particularly by capturing behavioral and metabolic domains not accounted for in conventional COPD indices.

Our findings demonstrated a negative correlation of LE8 and LC9 scores with all-cause death risk among COPD patients, while also revealing the independent contribution of PHQ-9 scores to COPD mortality. However, the C-index results indicated that incorporating depression scores into the LC9 metric demonstrated a marginally stronger association with outcomes compared with the LE8 score, although the magnitude of improvement was modest. We hypothesized that one explanation for this finding may be that the relationship between depression and other components of CVH attenuates the incremental predictive value gained by including depression. On one hand, depression can lead to poor dietary habits, reduced physical activity, smoking, and sleep disturbances, and can also trigger neurohumoral dysregulation and heightened inflammatory responses, which may subsequently elevate lipid, glucose, and blood pressure levels [18]. On the other hand, conditions such as obesity and sleep disorders have been shown to be connected with the development of depression among COPD patients [54]. Another possible explanation is that LC9 score is derived by combining LE8 score with PHQ-9 score. Because responses to the PHQ-9 are partially influenced by subjective interpretation [55], and the questionnaire emphasizes symptom frequency while placing limited emphasis on symptom intensity [56], LC9 score may not fully capture the contribution of depression relative to LE8 score. However, our study was only able to utilize the PHQ-9 to assess depression because of the constraints of the NHANES database. Therefore, future research designs are necessary to explore more comprehensive depression assessment metrics to enhance the predictive role of CVH in various diseases, including COPD.

This study’s primary advantages were its large sample size, prospective evaluation of COPD outcomes, and use of updated LC9 or LE8 scores to assess CVH. But there are also a few limitations that need to be noted. First, given that our study included only U.S. adults aged 40–79 years, the results should be generalized to other age groups or regions with caution. Therefore, further studies in more diverse populations are warranted. Secondly, despite accounting for a number of confounding variables, additional possible confounders, such as socioeconomic factors and COPD severity, which may simultaneously affect CVH scores and mortality risk, could not be fully accounted for. Future studies should collect additional potential confounding variables and apply quantitative methods, such as E-value analysis, for more precise evaluation. Thirdly, self-reported questionnaires were used to gather some CVH factors, which could lead to measurement and recall bias. Finally, the LC9, LE8 and PHQ-9 scores were calculated using only one-time point. This approach may not adequately capture chronic psychological burden. And disease progression, treatment, or acute exacerbation may alter behavioral patterns and depressive states during follow-up, while disease severity may suppress CVH scores, thereby creating a reverse causality relationship. The direction of such bias may attenuate the associations observed in this study. During the follow-up of the current survey, we could not account for anticipated lifestyle changes nor conduct precise lag analysis, which constitutes a crucial component of our future research plans.

In summary, this study identified a strong inverse relationship between all-cause mortality among COPD patients and CVH levels as measured by the LC9 and LE8 scores. Depression severity (PHQ-9) was positively correlated with all-cause death. The LC9 score’s predictive ability was marginally improved by adding depression scores to the LE8 framework, although this enhancement was minimal. Further research is needed to investigate extra predictive value of other mental health factors. These findings suggest that CVH indeed has significant potential as an excellent indicator for preventing COPD mortality, highlighting the significance of CVH levels in determining the prognosis of COPD patients. This could help guide more precise prevention strategies and targeted interventions to improve COPD patients’ overall care.

## Conclusion

Higher CVH levels defined by either LC9 or LE8 were strongly associated with reduced all-cause mortality in COPD patients. Taken together, the modest advantage of LC9 advocates for integrating psychological health into CVH evaluations to enhance risk prediction and optimize management strategies in COPD.

## Supplementary Information

Supplementary Material 1.

## Data Availability

Publicly available datasets were analyzed in this study. The full dataset can be accessed at: https://www.cdc.gov/nchs/nhanes/index.htm.
