# Supplementary material for: Comparing the predictive accuracy of life’s essential 8 and life’s crucial 9 scores for all-cause mortality in COPD patients among US adults: a prospective cohort study
Source: BMC Public Health. 2026 Jan 22;26:622. doi: 10.1186/s12889-026-26333-4 (PMC12911299; doi:10.1186/s12889-026-26333-4)

SUPPLEMENTARY MATERIALS

LIST

**Supplementary Tables**

Supplementary Table 1. Association between PHQ-9 score and all-cause mortality in patients with COPD.

Supplementary Table 2. Sensitivity analyses by excluding individuals with a follow-up time less than 24 months.

**Supplementary Figures**

Supplementary Figure1. The concordance index of LC9 score and LE8 score on all-cause mortality in patients with COPD.

**Supplementary Table**

**Supplementary Table 1. Metrics for measurement and quantitative assessment of CVH.**

| CVH metric | Method of measurement and Quantification of CVH metric | Scoring | |
| --- | --- | --- | --- |
| Diet | Self-reported daily intake of eating pattern: Quantiles of HEI-2015 | Points | Quantile |
|  |  | 100 | ≥95th percentile |
|  |  | 80 | 75th–94th percentile |
|  |  | 50 | 50th–74th percentile |
|  |  | 25 | 25th–49th percentile |
|  |  | 0 | 1st–24th percentile |
| Physical activity | Self-reported minutes of moderate or vigorous physical activity per week: Minutes of moderate- (or greater) intensity activity per week (NHANES PAQ-K questionnaire) | Points | Minutes |
|  |  | 100 | ≥150 |
|  |  | 90 | 120-149 |
|  |  | 80 | 90-119 |
|  |  | 60 | 60-89 |
|  |  | 40 | 30-59 |
|  |  | 20 | 1-29 |
|  |  | 0 | 0 |
| Nicotine exposure | Self-reported use of cigarettes or inhaled nicotine-delivery system: Combustible tobacco use or inhaled nicotine-delivery system use; or secondhand smoke exposure (NHANES SMQ) | Points | Status |
|  |  | 100 | Never smoker |
|  |  | 75 | Former smoker, quit ≥5 y |
|  |  | 50 | Former smoker, quit 1–<5 y |
|  |  | 25 | Former smoker, quit <1 y, or currently using inhaled nicotine-delivery system |
|  |  | 0 | Current smoker |
|  |  | Subtract 20 points (unless score is 0) for living with active indoor smoker in home | |
| Sleep health | Self-reported average hours of sleep per night: Average hours of sleep per night | Points | Level |
|  |  | 100 | 7–<9 |
|  |  | 90 | 9–<10 |
|  |  | 70 | 6–<7 |
|  |  | 40 | 5–<6 or ≥10 |
|  |  | 20 | 4–<5 |
|  |  | 0 | <4 |
| BMI | Body weight (kilograms) divided by height squared (meters squared): BMI (kg/m2) | Points | Level |
|  |  | 100 | <25 |
|  |  | 70 | 25.0–29.9 |
|  |  | 30 | 30.0–34.9 |
|  |  | 15 | 35.0–39.9 |
|  |  | 0 | ≥40.0 |
| Blood lipids | Plasma total and HDL cholesterol with calculation of non–HDL cholesterol: Non–HDL cholesterol (mg/dL) | Points | Level |
|  |  | 100 | <130 |
|  |  | 60 | 130–159 |
|  |  | 40 | 160–189 |
|  |  | 20 | 190–219 |
|  |  | 0 | ≥220 |
|  |  | If drug-treated level, subtract 20 points | |
| Blood glucose | FBG or casual HbA1c: FBG (mg/dL) or HbA1c (%) | Points | Level |
|  |  | 100 | No history of diabetes and FBG <100 (or HbA1c <5.7) |
|  |  | 60 | No diabetes and FBG 100–125 (or HbA1c 5.7–6.4) (prediabetes) |
|  |  | 40 | Diabetes with HbA1c <7.0 |
|  |  | 30 | Diabetes with HbA1c 7.0–7.9 |
|  |  | 20 | Diabetes with HbA1c 8.0–8.9 |
|  |  | 10 | Diabetes with HbA1c 9.0–9.9 |
|  |  | 0 | Diabetes with HbA1c ≥10.0 |
| Blood pressure | Appropriately measured systolic and diastolic blood pressures: Systolic and diastolic blood pressures (mm Hg) | Points | Level |
|  |  | 100 | <120/<80 |
|  |  | 75 | 120–129/<80 |
|  |  | 50 | 130–139 or 80–89 |
|  |  | 25 | 140–159 or 90–99 |
|  |  | 0 | ≥160 or ≥100 |
|  |  | Subtract 20 points if treated level | |
| Psychological health | Self-reported frequency of depression symptoms: NHANES PHQ-9 | Points | Level |
|  |  | 100 | 0-4 |
|  |  | 75 | 5-9 |
|  |  | 50 | 10-14 |
|  |  | 25 | 15-19 |
|  |  | 0 | 20-27 |

BMI indicates body mass index; FBG, cardiovascular health; FBG, fasting blood glucose; HbA1c, hemoglobin A1c; HDL, high-density lipoprotein; HEI, Healthy Eating Index; NDS, nicotine-delivery system; NHANES, National Health and Nutrition Examination Surveys; PAQ-K, Physical Activity Questionnaire K; SMQ, smoking assessment; and PHQ-9, Patient Health Questionnaire-9.

**Supplementary Table 2. Unweighted Cox regression analysis of Life’s Crucial 9 score for all-cause mortality in patients with COPD.**

|  | Crude Model | | Model 1 | | Model 2 | |
| --- | --- | --- | --- | --- | --- | --- |
|  | HR(95% CI) | *p* | HR(95% CI) | *p* | HR(95% CI) | *p* |
| LC9/10 | 0.81(0.75,0.87) | <0.0001 | 0.78(0.72,0.85) | <0.0001 | 0.81(0.74,0.89) | <0.0001 |
| Q1 [15.00, 48.33] | Ref |  | Ref |  | Ref |  |
| Q2 (48.33,58.33] | 0.87(0.67,1.13) | 0.30 | 0.74(0.57,0.96) | 0.03 | 0.80(0.61,1.05) | 0.11 |
| Q3 (58.33,67.78] | 0.64(0.49,0.85) | 0.002 | 0.59(0.44,0.79) | <0.001 | 0.66(0.48,0.89) | 0.01 |
| Q4 (67.78,97.22] | 0.44(0.32,0.59) | <0.0001 | 0.40(0.28,0.55) | <0.0001 | 0.46(0.32,0.67) | <0.0001 |
| p for trend |  | <0.0001 |  | <0.0001 |  | <0.0001 |

Crude Model: no covariates were adjusted.

Model 1: age, sex, race, marriage, education, and smoking status were adjusted.

Model 2: all covariates were adjusted.

COPD, Chronic Obstructive Pulmonary Disease; LC9, Life’s Crucial 9; HR: hazard ratio; 95% CI: 95% confidence interval; Q1-Q4: quartile 1-quartile 4.

**Supplementary Table 3. Unweighted Cox regression analysis of Life’s Essential 8 score for all-cause mortality in patients with COPD.**

|  | Crude Model | | Model 1 | | | Model 2 | |
| --- | --- | --- | --- | --- | --- | --- | --- |
|  | HR(95% CI) | *p* | HR(95% CI) | *p* | | HR(95% CI) | *p* |
| LE8/10 | 0.83(0.77,0.89) | <0.0001 | 0.83(0.77,0.90) | | <0.0001 | 0.87(0.80,0.95) | 0.002 |
| Q1 [11.25, 46.25] | Ref |  | Ref | |  | Ref |  |
| Q2 (46.25, 56.25] | 0.99(0.77,1.28) | 0.96 | 0.97(0.75,1.26) | | 0.81 | 1.03(0.79,1.34) | 0.83 |
| Q3 (56.25, 66.25] | 0.65(0.49,0.88) | 0.004 | 0.66(0.49,0.89) | | 0.01 | 0.77(0.56,1.06) | 0.11 |
| Q4(66.25,100.00] | 0.51(0.38,0.69) | <0.0001 | 0.53(0.38,0.73) | | <0.001 | 0.62(0.44,0.88) | 0.01 |
| p for trend |  | <0.0001 |  | | <0.0001 |  | 0.004 |

Crude Model: no covariates were adjusted.

Model 1: age, sex, race, marriage, education, and smoking status were adjusted.

Model 2: all covariates were adjusted.

COPD, Chronic Obstructive Pulmonary Disease; LE8, Life’s Essential 8; HR: hazard ratio; 95% CI: 95% confidence interval; Q1-Q4: quartile 1-quartile 4.

**Supplementary Table 4. Association between PHQ-9 score and all-cause mortality in patients with COPD.**

|  | Crude Model | | Model 1 | | Model 2 | |
| --- | --- | --- | --- | --- | --- | --- |
|  | HR(95% CI) | *p* | HR(95% CI) | *p* | HR(95% CI) | *p* |
| PHQ-9 score | 1.01(1.00,1.03) | 0.14 | 1.04(1.03,1.06) | <0.0001 | 1.04(1.01,1.06) | <0.001 |
| As a categorical variable using PHQ-9 ≥5 | | | | | | |
| [0, 4] | Ref |  | Ref |  | Ref |  |
| [5, 27] | 1.33(1.05,1.70) | 0.02 | 1.81(1.38,2.38) | <0.0001 | 1.70(1.28,2.27) | <0.001 |

Crude Model: no covariates were adjusted.

Model 1: age, sex, race, marriage, education, and smoking status were adjusted.

Model 2: all covariates were adjusted.

COPD, Chronic Obstructive Pulmonary Disease; PHQ-9, Patient Health Questionnaire-­9; HR: hazard ratio; 95% CI: 95% confidence interval.

**Supplementary Table 5. Sensitivity analysis of the association between Life’s Crucial 9 and all-cause mortality in patients with COPD based on a stricter definition of COPD.**

|  | Crude Model | | Model 1 | | Model 2 | |
| --- | --- | --- | --- | --- | --- | --- |
|  | HR(95% CI) | *p* | HR(95% CI) | *p* | HR(95% CI) | *p* |
| LC9/10 | 0.82(0.73,0.92) | <0.001 | 0.75(0.66,0.85) | <0.0001 | 0.77(0.66,090) | <0.001 |
| Q1 [15.00, 46.67] | Ref |  | Ref |  | Ref |  |
| Q2 (46.66,56.67] | 1.11(0.71,1.72) | 0.66 | 0.86(0.56,1.34) | 0.52 | 0.92(0.59,1.43) | 0.72 |
| Q3 (56.67,66.53] | 0.77(0.48,1.23) | 0.28 | 0.54(0.35,0.83) | 0.01 | 0.60(0.37,0.99) | 0.04 |
| Q4 (66.53,97.22] | 0.58(0.35,0.97) | 0.04 | 0.47(0.29,0.75) | 0.001 | 0.48(0.26,0.87) | 0.02 |
| p for trend |  | 0.01 |  | <0.001 |  | 0.02 |

Crude Model: no covariates were adjusted.

Model 1: age, sex, race, marriage, education, and smoking status were adjusted.

Model 2: all covariates were adjusted.

COPD, Chronic Obstructive Pulmonary Disease; LC9, Life’s Crucial 9; HR: hazard ratio; 95% CI: 95% confidence interval; Q1-Q4: quartile 1-quartile 4.

**Supplementary Table 6. Sensitivity analysis of the association between Life’s Essential 8 and all-cause mortality in patients with COPD based on a stricter definition of COPD.**

|  | Crude Model | | Model 1 | | | Model 2 | |
| --- | --- | --- | --- | --- | --- | --- | --- |
|  | HR(95% CI) | *p* | HR(95% CI) | *p* | | HR(95% CI) | *p* |
| LE8/10 | 0.83(0.73,0.94) | 0.003 | 0.79(0.70,0.89) | | <0.001 | 0.81(0.70,0.94) | 0.01 |
| Q1 [11.25, 44.38] | Ref |  | Ref | |  | Ref |  |
| Q2 (44.38, 54.38] | 0.77(0.52,1.15) | 0.20 | 0.70(0.47,1.05) | | 0.09 | 0.76(0.50,1.17) | 0.21 |
| Q3 (54.38, 64.38] | 0.71(0.45,1.12) | 0.14 | 0.60(0.39,0.93) | | 0.02 | 0.69(0.41,1.16) | 0.16 |
| Q4 (64.38,96.88] | 0.51(0.31,0.83) | 0.01 | 0.47(0.29,0.76) | | 0.002 | 0.55(0.31,0.98) | 0.04 |
| p for trend |  | 0.01 |  | | 0.002 |  | 0.05 |

Crude Model: no covariates were adjusted.

Model 1: age, sex, race, marriage, education, and smoking status were adjusted.

Model 2: all covariates were adjusted.

COPD, Chronic Obstructive Pulmonary Disease; LE8, Life’s Essential 8; HR: hazard ratio; 95% CI: 95% confidence interval; Q1-Q4: quartile 1-quartile 4.

**Supplementary Table 7. Analysis of the association between Life’s Crucial 9 / Life’s Essential 8 and all-cause mortality risk in COPD stratified by age and metabolic syndrome.**

|  | LC9 | | LE8 | |
| --- | --- | --- | --- | --- |
| Characteristics | HR(95% CI) | *p* | HR(95% CI) | *p* |
| Age group |  |  | *p* for interaction = 0.19 | |
| 40-49 | 0.95(0.92,0.99) | 0.008 |  |  |
| 50-59 | 0.95(0.93,0.97) | <0.0001 |  |  |
| 60-69 | 0.97(0.95,0.98) | <0.0001 |  |  |
| 70-79 | 0.98(0.97,0.99) | <0.0001 |  |  |
| MetS-ATP |  |  |  |  |
| Yes | 0.99(0.97,1.00) | 0.086 | 0.99(0.97,1.01) | 0.166 |
| No | 0.96(0.95,0.98) | <0.0001 | 0.97(0.95,0.98) | <0.0001 |

Note: This table presents stratified analysis results, showing only subgroup-specific effects, which are consistent with the p for interaction derived from the likelihood ratio tests reported in the main text.

COPD, Chronic Obstructive Pulmonary Disease; LC9, Life’s Crucial 9; LE8, Life’s Essential 8; HR: hazard ratio; 95% CI: 95% confidence interval.

**Supplementary Table 8. Sensitivity analyses by excluding individuals with a follow-up time less than 24 months.**

|  | Crude Model | | Model 1 | | Model 2 | |
| --- | --- | --- | --- | --- | --- | --- |
|  | HR(95% CI) | *p* | HR(95% CI) | *p* | HR(95% CI) | *p* |
| **LC9/10** | 0.77(0.70,0.86) | <0.0001 | 0.74(0.67,0.82) | <0.0001 | 0.78(0.68,0.90) | <0.001 |
| Q1 | Ref |  | Ref |  | Ref |  |
| Q2 | 1.08(0.72,1.63) | 0.69 | 0.95(0.64,1.42) | 0.82 | 1.05(0.69,1.62) | 0.81 |
| Q3 | 0.70(0.44,1.11) | 0.13 | 0.58(0.38,0.89) | 0.01 | 0.69(0.44,1.09) | 0.11 |
| Q4 | 0.40(0.25,0.66) | <0.001 | 0.35(0.22,0.56) | <0.0001 | 0.44(0.25,0.79) | 0.01 |
| *p* for trend |  | <0.0001 |  | <0.0001 |  | <0.001 |
| **LE8/10** | 0.78(0.70,0.87) | <0.0001 | 0.77(0.70,0.86) | <0.0001 | 0.82(0.72,0.93) | 0.003 |
| Q1 | Ref |  | Ref |  | Ref |  |
| Q2 | 0.81(0.55,1.21) | 0.30 | 0.84(0.58,1.21) | 0.35 | 0.95(0.65,1.39) | 0.78 |
| Q3 | 0.69(0.42,1.13) | 0.14 | 0.66(0.43,1.00) | 0.05 | 0.82(0.50,1.32) | 0.41 |
| Q4 | 0.37(0.23,0.58) | <0.0001 | 0.36(0.23,0.58) | <0.0001 | 0.46(0.26,0.81) | 0.01 |
| *p* for trend |  | <0.0001 |  | <0.0001 |  | 0.01 |

Crude Model: no covariates were adjusted.

Model 1: age, sex, race, marriage, education, and smoking status were adjusted.

Model 2: all covariates were adjusted.

COPD, Chronic Obstructive Pulmonary Disease; LC9, Life’s Crucial 9; LE8, Life’s Essential 8; HR: hazard ratio; 95% CI: 95% confidence interval; Q1-Q4: quartile 1-quartile 4.

**Supplementary Table 9. Sensitivity analyses by excluding individuals with a follow-up time less than 36 months.**

|  | Crude Model | | Model 1 | | Model 2 | |
| --- | --- | --- | --- | --- | --- | --- |
|  | HR(95% CI) | *p* | HR(95% CI) | *p* | HR(95% CI) | *p* |
| **LC9/10** | 0.77(0.68,0.87) | <0.0001 | 0.74(0.65,0.84) | <0.0001 | 0.78(0.67,0.91) | 0.002 |
| Q1 | Ref |  | Ref |  | Ref |  |
| Q2 | 1.04(0.68,1.60) | 0.86 | 0.95(0.63,1.44) | 0.82 | 1.07(0.70,1.64) | 0.75 |
| Q3 | 0.62(0.38,1.00) | 0.05 | 0.53(0.34,0.82) | 0.004 | 0.63(0.40,0.99) | 0.04 |
| Q4 | 0.42(0.26,0.69) | <0.001 | 0.38(0.23,0.62) | <0.001 | 0.48(0.26,0.88) | 0.02 |
| *p* for trend |  | <0.0001 |  | <0.0001 |  | 0.004 |
| **LE8/10** | 0.78(0.69,0.88) | <0.0001 | 0.77(0.68,0.87) | <0.0001 | 0.82(0.70,0.95) | 0.01 |
| Q1 | Ref |  | Ref |  | Ref |  |
| Q2 | 0.81(0.52,1.27) | 0.37 | 0.87(0.56,1.32) | 0.50 | 0.96(0.62,1.48) | 0.85 |
| Q3 | 0.61(0.35,1.06) | 0.08 | 0.60(0.37,0.97) | 0.04 | 0.72(0.42,1.24) | 0.23 |
| Q4 | 0.39(0.24,0.65) | <0.001 | 0.40(0.24,0.67) | <0.001 | 0.51(0.27,0.95) | 0.03 |
| *p* for trend |  | <0.001 |  | <0.001 |  | 0.02 |

Crude Model: no covariates were adjusted.

Model 1: age, sex, race, marriage, education, and smoking status were adjusted.

Model 2: all covariates were adjusted.

COPD, Chronic Obstructive Pulmonary Disease; LC9, Life’s Crucial 9; LE8, Life’s Essential 8; HR: hazard ratio; 95% CI: 95% confidence interval; Q1-Q4: quartile 1-quartile 4.

**Supplementary Figure**

**Supplementary Figure1. The time-dependent AUC curves of Life’s Crucial 9 and Life’s Essential 8 on all-cause mortality in patients with COPD.**


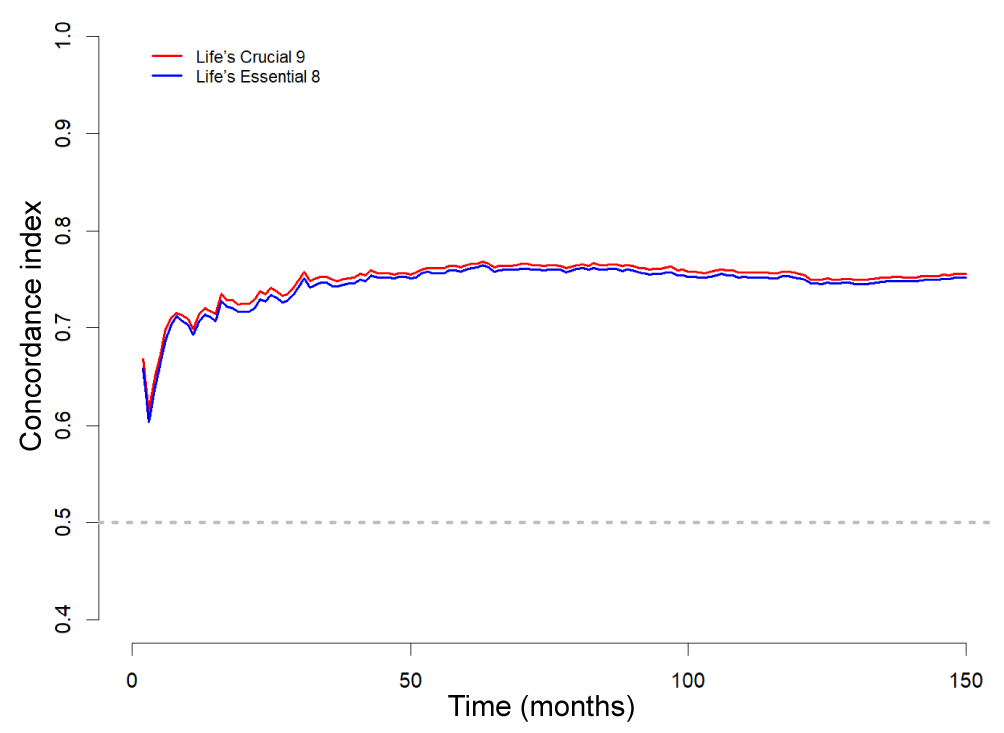

Supplement: Supplementary file 1 — Supplementary Material 1. [file 12889_2026_26333_MOESM1_ESM.docx]
